# Synthetic Phage for Tissue Regeneration

**DOI:** 10.1155/2014/192790

**Published:** 2014-05-27

**Authors:** So Young Yoo, Anna Merzlyak, Seung-Wuk Lee

**Affiliations:** ^1^Convergence Stem Cell Research Center, Medical Research Institute, Pusan National University School of Medicine, Yangsan 626-870, Republic of Korea; ^2^Department of Bioengineering, University of California, Berkeley, and Physical Bioscience Division, Lawrence Berkeley National Laboratory, Berkeley, CA 94720, USA

## Abstract

Controlling structural organization and signaling motif display is of great importance to design the functional tissue regenerating materials. Synthetic phage, genetically engineered M13 bacteriophage has been recently introduced as novel tissue regeneration materials to display a high density of cell-signaling peptides on their major coat proteins for tissue regeneration purposes. Structural advantages of their long-rod shape and monodispersity can be taken together to construct nanofibrous scaffolds which support cell proliferation and differentiation as well as direct orientation of their growth in two or three dimensions. This review demonstrated how functional synthetic phage is designed and subsequently utilized for tissue regeneration that offers potential cell therapy.

## 1. Synthetic Phage

**Figure 1:**
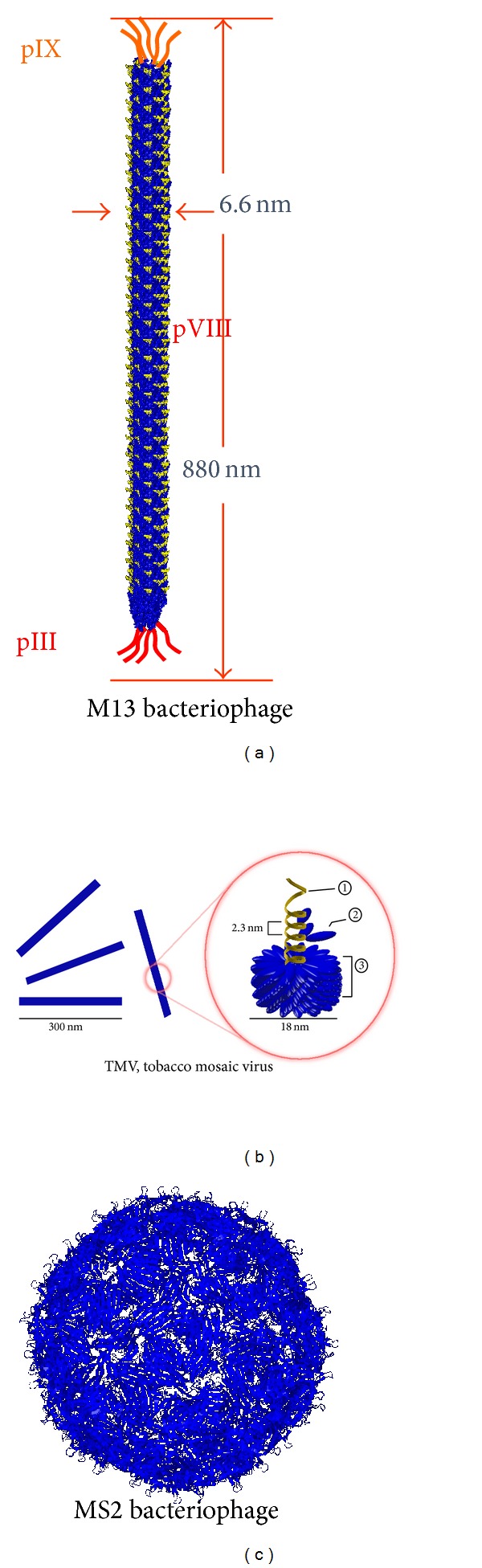
Schematic diagram of various distinct structures of various phages. (a) Long rod structure of M13 bacteriophage with genomic schematic diagrams to show each protein expressed on the M13 phage surfaces. (b) Structure of Tobacco mosaic virus, a rod-like structured plant virus, made of single strand RNA *①* and capsid *③* composed of coat *②* proteins. (c) Sphere structure of MS2 bacteriophage.

**Figure 2:**
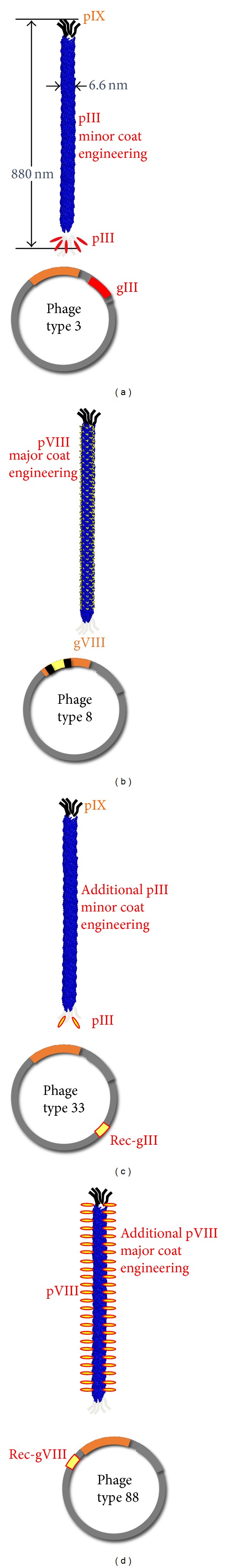
Multifunctional synthetic phage construction. (a) Type 3 phage engineering, (b) Type 8 phage engineering, (c) Type 3 + 3 phage engineering, (d) Type 8 + 8 phage engineering.

The meaning of synthetic, as defined in a dictionary, is “combination, composition, putting together, opposite of analysis; building up of separate elements especially of conception or propositions or facts, into a connected whole.” Combined with biology, it became synthetic biology meaning engineering complex living systems through novel assemblies of biological molecules. Synthetic biology has been paved after recombinant DNA technology in the 1970s. So originally synthetic means recombinant or engineered, in biology. Although useful engineering system based on T4, T7, *λ*, or other viruses has been introduced, the technology is mostly fully developed in the filamentous phages (Figure 1). Phages are viruses that infect bacterial cells, and many commercial vectors for recombinant DNA studies are from phages. Synthetic phage utilizes their genetic information of coat proteins. Phage display is a practical example of how we can make synthetic viruses for different purposes. Using standard recombinant DNA technology, interested foreign peptides (or proteins) expression can be realized by incorporation of corresponding coding sequences onto each replicable viral coat DNA (Figure 2). Useful peptides identified or engineered by synthetic phages were summarized in Table 1.

### 1.1. Genetic Engineering of Phage: pIII Minor Coat or pVIII Major Coat Protein Engineering

pIII minor coat protein engineering (Type 3) is relatively well known for insertion of foreign peptides. Foreign peptides displayed on all five pIII subunits are constrained to lie very close to each other, but their attachment to the virion surface is probably quite flexible. For these reasons, it is likely that such displayed peptides can form multivalent interactions with immobilized selectors or cellular receptors. Phage display has been developed for use as information mining tools [1–3], in which the diversity of the amino acid libraries presented by the phage gives the binding information between the peptide and its target [4–6]. Recently, the synthetic phage displaying Fab in pIII region was utilized as in phage display and expanded its capacity of expressing peptide sizes and their selectivity and sensitivity [7]. Taken together, phage display was used to identify the peptides mimicking many functional peptides including chemokines or chemokine receptors, which was then utilized for studying or targeting the role of chemokines and receptors [8–11] (Also see Table 1).

Lee group [12–14, 16, 27] mostly utilized pVIII engineering for tissue engineering purposes but also did pIII engineering for making multifunctional synthetic phages on sensing or capturing purposes (Table 1). Foreign peptides display on pVIII was introduced soon after pIII display was introduced. The “landscape” peptide presentation on the major coat protein of the filamentous phage has been utilized to template inorganic crystals for energy and memory storage devices [28–32] and make stimulus responsive materials [33]. The phage has also been exploited for medical applications, such as targeted drug [34–36], gene [37], imaging agent [38] delivery, and a tissue engineering scaffold material [12]. Merzlyak et al. presented a cell signaling RGD motif on pVIII proteins for a tissue engineering scaffold application [12, 27]. The approach which is used to display a foreign peptide on every copy of pVIII protein demonstrated how other functionally designed groups can be presented on the phage filament with quantitative analyses on the characteristics of the inserts and their constrained sequences expressed on a phage particle.

### 1.2. Genetic Engineering of Phage: pVI, pVII, or pIX Minor Coat Protein Engineering

Other minor coat proteins, pVI, pVII, or pIX, have been used in phagemid format, which will have mosaic display of inserted foreign peptides [39, 40]. Fusion proteins were expressed on pVII and pIV from the phagemid as procoats with* omp*A and* pel*B leaders. Since these proteins are likely to interact with one another in the phage capsid, the method may be useful to engineer antibodies or intergrins which are dimeric proteins. This technology was later extended to construct a large, human single-chain Fv (scFv) antibody library on pIX [41].

### 1.3. Genetic Engineering of Phage: NN Type Engineering

A mosaic display using type 88 or 33 systems overcomes two potential disadvantages of pVIII major coat modification (Type 8) and pIII minor coat modification [42, 43]. The type 88 vectors contain synthetic recombinant gene pVIII beside wild pVIII genes. To minimize recombination between the recombinant and wild type pVIII genes, the sequence of the recombinant pVIII gene is designed to be very different from the wild type pVIII gene, while encoding the same amino acid sequences. Similarly, type 33 system has two pIII genes of one full length and one truncated (amino acids 198–408). The former expresses a functional pIII, while the second gene produces a fusion protein.

## 2. Synthetic Phage for Tissue Regeneration

Tissue engineering scaffolding materials are ultimately designed to imitate the extracellular matrix (ECM), a fibrous protein network that houses the cells* in vivo*. This network provides cells with physical support and guidance through a specific topographical and chemical presentation of various adhesive sites and growth factors. Therefore, in order to control cellular behaviors such as adhesion, proliferation, and differentiation within the man-made scaffolds, their surface functionalization with bioactive molecules is highly desirable [44–46]. Furthermore the control over density of such bioactive groups [47–49] and their geometric patterning [47, 50, 51] has been shown important in biomaterials' ability to modulate such behaviors. Majority of current fabrication methods rely on chemical processing to functionalize biomaterials. With this method the final density of bioactive groups presented on the surface is ultimately dictated by the bulk solution concentration [45, 46, 52]. The local binding properties of the material surface, such as charge or availability of reactive groups or receptors, dictate the final spacing of the bioactive groups. Most techniques that allow for a very precise micro- and nanoscale chemical patterning of a substrate are lithography based (i.e., dip-pen lithography) and are hard to replicate in large scale or in three-dimensional scaffold materials [44, 50]. Recently developed nanofabrication techniques, such as peptide self-assembly, electrospinning, and polymer phase-separation, come closer to mimicking the natural ECM topographically; however, the controlled presentation of single or multiple functional groups is still lacking [44, 48, 49].

**Figure 3:**
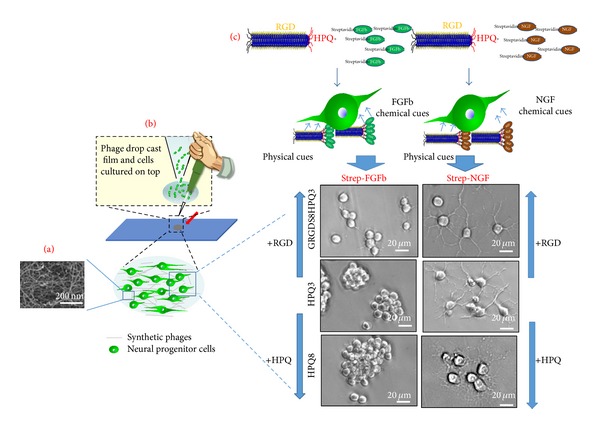
Phage based tissue engineering materials. (a)-(b) ECM-like nanofibrous structured phage network (SEM image) can be made by drop cast film. (c) Neural progenitor cells cultured on top of synthetic phages responded to the growth factor immobilized by HPQ-phages via streptavidin. Physical and chemical cues provided by synthetic phages could control cellular behaviors [14].

Viruses are some of the best characterized structurally organized large molecules. Their nanoscale size and inherent monodispersity of their shape and surface chemistry are better than what can be achieved with most synthetic nanoparticles to date [53]. Both genetic and chemical pathways have been used to modify either single or multiple virus capsid proteins with functional groups [28, 37, 47, 53–55]. Moreover novel binding ligands can be found through evolutionary phage display screening methods [2, 3, 5]. Such functionalized virus particles have been demonstrated to selectively bind both inorganic and organic particles. Additionally the templation of virus particles has been utilized for electronic and magnetic materials [28, 56, 57], as well as a variety of medical applications [34–36, 38]. M13 bacteriophage is a filamentous bacterial virus. It has a defined long-rod shape at 880 nm long and 6.7 nm in diameter, with precisely positioned major and minor capsid proteins. These coat proteins can be genetically engineered to express short peptide groups [28, 56, 58]. M13 has been previously genetically engineered phage to display cell-adhesive peptides such as RGD and IKVAV on every copy of its pVIII protein [12]. Furthermore such modified filamentous phage for construction of aligned two- and three-dimensional materials that are able to support and control the polarization of cells such as fibroblasts and neural progenitor cells was demonstrated [12]. Chimeric displays of binding groups on M13 phage have been demonstrated previously for drug delivery [37, 59], ELISA [60], and electronic [56] applications. Additional engineering of the M13 phage to express biotin-like HPQ motifs on their capsid proteins will allow for a functional expansion of potential scaffold interactions with the cells, as it will be able to present a variety of immobilized avidin conjugated growth factors and cytokines. Unique biochemical and structural features of genetically engineered phage can be also used in the context of tissue engineering in order to control cellular growth or differentiation (Figure  3, [14]).

### 2.1. Chemical Cue Control by Synthetic Virus

Merzlyak et al., for example, have explored the use of genetically modified M13 phage as a novel building block for neural cell engineering materials to make functional biomaterials for tissue regeneration by chemical cue control [12]. This was accomplished by engineering the phage to display specific cell signaling motifs and then assembling the viral particles into a macroscopic scaffolding material. Many peptide expression systems have previously been demonstrated on the various capsid proteins of the phage through creation of peptide libraries [3, 42]. However, as a biological particle for peptide display, phages possess the inherent limitation of having to be successfully expressed and assembled within the* E. coli* bacteria host, which restricts the type and number of peptides that can be displayed [61–64]. They developed a novel cloning approach for display of an integrin-binding RGD motif on every copy of the pVIII major coat protein [12]. The researchers constructed the phage using a partial library, in which an engineered octamer insert for pVIII included a constrained RGD group that was surrounded by flanking degenerate residues. This allowed for expression of inserts that retained the desired function of the RGD motif and yet were biologically compatible with* E. coli* during the intricate phage replication process. After construction of engineered phage that stably displayed either RGD- or IKVAV-peptide groups on every copy of the pVIII protein, they constructed aligned two- and three-dimensional scaffolding materials containing phage and tested their applicability for tissue engineering. Biocompatibility of the synthetic phage materials was tested by growing NIH-3T3 fibroblast and neural progenitor cells on phage films and in phage containing media [12, 65]. Both cell types showed normal morphology and proliferation when in direct contact with phage materials. Neural progenitor cells either retained their progenitor state or differentiated towards the neural cell phenotype depending on media conditions. It was then demonstrated that three-dimensional phage materials could support proliferation and differentiation of neural progenitor cells. Both RGD- and IKVAV-phage matrices facilitated colony formation of neural progenitor cells, which sustained a viability of over 85% during the seven-day observation period. In comparison to RGE and wild type phage controls, RGD and IKVAV phage resulted in enhanced binding and spreading of neural progenitor cells with high specificity. Finally, by simple extrusion or spinning of phage solution, the researchers constructed aligned three-dimensional phage fiber matrices with embedded neural progenitor cells. The resulting phage fibers encouraged neural cell differentiation and directed cell growth parallel to the long axis of the fibers [12]. Chung et al. showed mechanical shearing of phage solution on a glass substrate which resulted in two-dimensional directionally oriented films. These oriented films were shown to direct the alignment and morphology of fibroblasts, osteoblasts, and neural cells [65].

### 2.2. Physical Cue Control by Synthetic Virus

Studies on chemical cue and physical cue provided by synthetic phages were performed with the RGD- and DGEA-peptides engineering phage films and fibers. Yoo et al. demonstrated the early osteogenic differentiation of mouse preosteoblasts by using collagen-derived DGEA-peptide on nanofibrous phage tissue matrices [16]. They constructed major coat engineered with DGEA, DGDA-, or EGEA-peptides. By genetic engineering of phages, they could construct nanofiber-like shaped phages having 2700 copies of the target peptides from the inserted genes with 2 and 2.7 nm spacing laterally and axially, respectively. By constructing the phage-based tissue matrix systems, they could investigate the specific effect of biochemical cues, which can be tuned precisely at a single amino acid level with little change in other physical and chemical properties. They characterized the chemical cue or physical cue effects of DGEA- and of RGD-peptides on the synthetic M13 phage backbone by applying MC3T3 preosteoblast cells on fabricated phage 2D film and 3D fibers. They could observe pronounced outgrowth of the preosteoblast on DGEA-phage matrices. The cells are spread very well throughout the samples on the DGEA-phage matrices. Cells on DGDA, EGEA, or RGE-phages, which are different in one single amino acid from DGEA- or RGD-phages, showed that the responses are DGEA peptide-specific, in which synthetic phage-based chemical cues can be controlled by genetic engineering. Competition assay with corresponding peptide with the engineered phage confirmed that the peptide specific chemical cues were controlled by synthetic phage. The DGEA-peptide specific outgrown morphology of preosteoblasts forms on the 2D cultures phage matrices, which were also observed in 3D cultures. In addition, the DGEA-specific morphological responses of preosteoblast cells are linked with early osteogenic differentiation by DGEA-peptides.

Virus structure can give more effective and efficient physical cues. The self-assembly capabilities of phage with patterning techniques can enhance the phages' specific biochemical and physical cues. Yoo et al. developed a facile patterning method of patterning genetically engineered M13 bacteriophage by employing microcontact printing methods to provide human fibroblast cells with specific biochemical and physical cues [13]. They demonstrated that nanofibrous structures, along with the biochemical signals presented by the phage microstructures, are critical to guide cellular growth and morphologies. The enhanced cellular morphological responses to RGD-phage topology rather than to RGD-peptide itself show that phage nanofibrous structure contributes in controlling physical cues. Especially rod-like viruses such as M13 and TMV can control their physical cues and mechanical cues even only by their concentration. Lin et al. reported the formation of diverse patterns which resulted from drying a solution of rod-like TMV particles in a glass capillary tube [66]. The concentration of TMV, the salt concentration in aqueous solution, and the surface properties of the capillary tube interior were used as three key factors to govern such combined self-assembly behavior. The formation of hierarchical structures which can be again used for guiding directional cellular growth was determined by the preferred orientation of TMV at the air-liquid interface as well as the pinning-depinning process. By controlling the key factors, they could generate the surface roughness together with patterned structure, which was then used for rat aortic smooth muscle cell (SMC) culture for the direct orientation of cells. They could finally generate either stress-induced SMC alignment or 2D patterns by utilizing the TMV patterns.

### 2.3. Multifunctional Phage Materials

The physiological cellular environments present a variety of cell signaling motives simultaneously including adhesive sites, growth factor, and other cytokine molecules to influence the cellular behavior [46, 67–69]. Similarly engineering materials incorporating several signaling motives simultaneously have shown this synergy to be more effective than single motives alone [46, 48, 68, 70]. For example, a study by Dr. Jeffrey Hubbell's group demonstrated that the incorporation of several functional peptide groups derived from the laminin into a fibrin matrix at the same time resulted in a synergistic effect on cell differentiation. The cell neurites were extended further in the peptide combination matrix then predicted by just an additive effect from each peptide's contribution [48]. Immobilization of growth factor molecules to the matrix surface, instead of their untethered encapsulation within it, can decrease the uncontrolled release of these molecules, as well as their internalization and metabolization by the cells, and therefore provide the cells with a more directed and sustained signal, further influencing their behavior [46]. Multiple chemical cue controls can be provided by using M13 synthetic phage system. Yoo et al. developed a facile growth factor immobilization system by utilizing multiple functionalized M13 synthetic phage based matrices [14]. The immobilized growth factor by M13 synthetic phage, together with phage's nanostructure itself, can give simplified cellular environment which actually consists of signaling motifs, growth factors, and topological structure effects. Synthetic phage based system shows its advantage for providing multifunctional chemical cues. Multiple signalling and therapeutic peptide motifs can be simultaneously displayed on the pIII, pVIII, and pIX protein coats of M13 phages through genetic modification [3, 42]. They constructed His-Pro-Gln (HPQ) peptide either on pVIII or on pIII phage coat proteins. The HPQ motif allows binding to streptavidin-conjugated molecules, so that streptavidin-conjugated growth factor can be immobilized without any size limitation decorating on M13 phage coat protein. This facile growth factor immobilization approach by synthetic phage may be useful for studying biochemical cues in cell biology and also creating tissue engineering materials. Through the HPQ sites, they were able to immobilize streptavidin-conjugated FGFb and NGF onto phage matrices. They also modified RGD peptide, which is well known to promote cell adhesion and well distribution of cells, on major coat proteins. They demonstrated that the growth factors immobilized on the multifunctionalized M13 phage matrices with HPQ- and RGD-peptides were functional and could direct cell growth towards desired cellular morphologies by RGD peptide and towards cellular fate, FGFb for proliferation and NGF for differentiation (Figure 3).

With the phage particle modular with an HPQ motif, a variety of factors can be immobilized on the phage matrix, correspondingly influencing different cell behaviors or even different cell types. For example, EGF factor can be immobilized on the phage to induce differentiation of the progenitor cells to the neuronal phenotype [46]. Similarly a bone morphogenic protein (BMP) and insulin growth factor (IGF) can be immobilized to assist in the differentiation of osteoblast cells [70]. Furthermore vascular endothelial growth factor (VEGF) can be immobilized on the matrix to enhance endothelial cell adhesion for vascular tissue engineering [71]. Several excellent recent reviews describe the function of many biologically relevant short peptide groups, growth factors, and cytokines [46, 68, 70, 71]. Additionally as vascular cells are aligned in their native environment the alignment capabilities of the phage matrices could be further beneficial for their defined directional growth. If needed even further functionalization of the phage can be accomplished by various chemical conjugation schemes, which have recently been employed in modifying other virus particles [53, 55]. After the design and engineering of the individual phage macromolecules, their various ratios can be mixed into a homogenous solution at different concentrations to further explore how molecular concentration gradients can influence cellular behavior* in vitro* models [72]. After such systematic analysis the design parameters that work best can be incorporated into a final mix solution to be tested on the* in vivo* systems.

## 3. Immune Study and Therapeutic Applications of Phage Materials

As the phage material we discussed is ultimately designed for* in vivo* applications, synthetic phage based future works will explore both* in vitro* and* in vivo* immunogenic responses to the phage matrices. We hypothesize that the phage matrix as a foreign protein mass will be recognized as a “non-self” material, via the complement system [73]. In the immune privileged environment of the central nervous system, microglia, specialized immune cells of the brain, will likely mediate the immune response [38, 74]. Previous studies have seen no inflammation related damage at the phage targeted tissue site [38]. However, if the greater concentration of the phage activate the microglia, their recruitment to the site of injury may actually facilitate nerve tissue regeneration by clearance of cellular and ECM debris of the glial scar and expression of the growth factors and the native extra cellular proteins, such as laminin [75]. To explore a similar mechanism of action there is currently a phase II clinical trial study to test the efficacy of injecting macrophage cells to the site of spinal injury on stimulating regeneration [50].* In vitro* immunogenic studies will be conducted to assess the potential of phage materials to induce an immunogenic inflammation reaction. Similar to a study conducted by Ainslie et al. testing the inflammation reaction of nPTFE material [76], a panel measuring the level of immuno stimulating or inhibiting cytokines can be performed on the supernatant from the macrophage cells grown on the phage substrates. Tissue culture polystyrene can serve as a negative control, and macrophages stimulated by lipopolysaccharides as a positive control. The levels of cytokines present can be assessed for their immunostimulating and immunoinhibiting activity. If very high levels of immunostimulating molecules such as IL-1 or TNF-*α* are noted, phage may be modified to express compliment inhibiting peptides [77, 78]. Furthermore, as was done in a study by Silva et al.,* in vivo* studies can be performed by injecting phage solution into spinal cord area of rat animal subjects [49]. Following the injection the behavior of the animals can be evaluated for changes. After the sacrifice of animal subjects injection site can be evaluated with histological studies to evaluate for tissue inflammation and fibrosis. A previous study that targeted engineered phage solution to a *β*-amyloid plaques in the brain did not see any adverse tissue reactions with histological analysis [38].

### 3.1. Mechanical and Degradation Properties of Phage Matrices

Control of mechanical and degradation properties of the biomaterials is important for tissue engineering applications. In an optimal engineering scenario the material that is intended to replace or repair a tissue will remain at the site of injury until it is remodeled by the cells and replaced by the naturally produced ECM [68, 79]. Previous work with hydrogels has demonstrated that both the concentration of the polymer macromolecule units and the degree of their crosslinking can be used to tune the mechanical properties and the rate of degradation of these materials [68, 79]. Lee group encapsulated the phage materials in an agarose gel to keep them stable in the media solution over the course of experiment [12, 16]. A future project that can further improve upon the phage scaffolds is to increase their stability in aqueous media environments. Preliminary work conducted in our lab on crosslinking chemically biotinylated phage with streptavidin shows a much improved stability of the phage fibers, which remain in solution for over a week without degradation [80].

### 3.2. Gene Delivery System

Drug delivery and tissue engineering materials are often very closely related in both function and architecture. In fact there is one perspective in the scientific community that tissue engineering scaffolds are just a delivery system of cells into the body [45]. Additionally the lines between the two areas get blurred when controlled growth factor or cytokine release is incorporated into the matrix to influence either the contained or the surrounding cells [45, 46, 70, 79]. By the streptavidin crosslinking methods described above small therapeutic drug molecules may be incorporated into the matrix. Furthermore the link to the phage can be engineered to be dependent on enzymatic cleavage [68, 81] so that the delivered molecules are released only when they are sequestered by the cell activity. Therapeutic genetic material can be incorporated into the phage DNA and carried within the phage capsule for specific delivery to the cells via receptor uptake [37]. As the M13 phages are nonlytic, they will be continuously produced by the bacteria without causing bacterial wall rupture or the resulting debris. By designing the peptide expression on the phage capsid they can be more locally targeted to cell receptors (i.e., via RGD or other ligands). Phage display technology has allowed for identification of novel homing peptides that target unknown cell surface proteins. The targeting peptides can be incorporated into bacteriophage coat proteins through the genetic engineering techniques described previously [37]. These include peptides (RGD, glioma-binding peptide) [82, 83], HER2 receptor targeting antibody [84], growth factors (EGF, FGF2) [85–87], and the penton base of adenovirus [88]. Similar to drug delivery, nucleic acid materials are now being incorporated in the scaffolding materials for delivery to the cells. Furthermore it has been shown that DNA materials that are tethered to the matrix, rather than just encapsulated are more effectively transferred to the cell [89]. Phage particles engineered as described above to contain the genetic load for cell delivery as well as specific cell targeting peptides can be cross-linked with streptavidin units to produce stable tissue engineering scaffolds. As these scaffolds get taken up and degraded by cell endocytosis [90], the phage could release their gene cargo and further induce cell behavior.

### 3.3. Diagnosis and Therapeutic Application

Thanks to phage display technology, we could find various useful peptide information which can be developed further for imaging and diagnosis of certain diseases, such as cancer [91]. For the therapeutic application, antibody phage display has been developed and being tested for clinical approval [92]. Another application study of utilizing M13 synthetic phage properties by adopting different useful virus parts was also introduced. Hajitou et al. constructed hybrid phage with two genes from phage and nucleus integrating gene from AAV, called inverted terminal repeats. Additionally, these phages were engineered integrin binding peptide on minor coat proteins. Therefore, the RGD peptide induced internalization of the phage through integrin mediated endocytosis process and the inverted terminal repeats (ITR) led to improved transgene expression, which is linked to functioning of delivered gene, in the cytoplasm. The resulting AAV/phage system provided superior tumor transduction over phage alone. Topical delivery by applying these therapeutic synthetic phage materials onto localized disease areas with specific integrating functions might reduce the risk of the side effects and enhance the efficiency of the drug delivery.

## 4. Summary and Future Perspectives

In this paper, we have majorly explored the use of M13 bacteriophage (phage) as a novel building block together with providing specific functions for tissue engineering materials. Prior to using it as a biomimetic tissue engineering scaffold material, the phage was decorated with cell signaling motifs. An incredible diversity of peptide expression has previously been demonstrated on the various capsid proteins of the phage through creation of peptide libraries [1–3]. A novel cloning approach to display an integrin binding RGD motif on every copy of pVIII was introduced to decorate the phage, the major coat protein of M13 phage. Merzlyak et al. did this by using a partial library method, where an engineered octamer is inserted for the pVIII including a constrained RGD group surrounded by a degenerate residue library. This allowed the expression of full inserts that retained the desired RGD motif yet were favorably compatible with all the protein interactions inherent in phage replication process within* E. coli*. Furthermore they systematically analyzed the allowed amino acid sequence space for pVIII inserts by making constrained libraries with chemically variable residues (positive, negative, and hydrophobic) [12]. This approach can be useful for engineering phage particles with a very dense uniform display of short signal peptide motifs that may be beneficial for tissue engineering materials [12, 48, 49, 93]. After demonstrating the phage as an able filament particle to form aligned scaffolds that are both conducive and instructive to cell growth, further phage design improvements by making it multifunctional can be made. The phage was engineered to incorporate an adhesive peptide motive RGD on pVIII and a constrained biotin-like HPQ motif on pIII protein [14, 37, 94]. There are limits in the ability of the phage to display a multivalency of protein molecules based on the size and sequence of the insert [3], and it cannot be altered via genetic means to present functional carbohydrate molecules. By exploiting the binding affinity of biotin-streptavidin bond, with an engineered biotin-like HPQ group we imparted a modular functionality to the phage building block [14]. Any growth factor, cytokine, or an otherwise therapeutic molecule conjugated to an avidin will be able to bind to our engineered HPQ phage and further functionalize the matrix [14, 95].

In summary, we have introduced the utilization of genetically engineered M13 bacteriophage (synthetic phage) as a functional building block for tissue engineering matrices that can guide adhesion, polarization, and alignment behavior of cells. We have also presented a number of avenues that can be used to expand this area of research further to immune/chemokine study and use phage for highly functional and useful biomaterials to the potential application area of diagnosis and therapy.

## Figures and Tables

**Table 1:** Selected reports of peptides identified or constructed by synthetic phages.

Peptide sequence	Engineering type	Targeted protein	Biological activity	Potential applications	References
RGD	3 and 8	Integrin *α*_*V*_ *β*_1_	Cellular adhesion, fibronectin like	Tissue engineering, regeneration, receptor-mediated endocytosis	[12–15]

IKVAV	8	Integrin *α*_6_ *β*_4_	Cellular adhesion, laminin like	Tissue engineering, neural cell differentiation	[12]

DGEA	8	Integrin *α*_2_ *β*_1_	Cellular adhesion, collagen type I like	Tissue engineering, osteogenic differentiation	[16]

HPQ	3 and 8	Streptavidin	Streptavidin binding, biotin like	Tissue engineering, conjugating growth factors	[14, 17]

RLIVGDPSSFQEKDADTL	3	Chlamydia	Ameliorating chlamydia infection	Prevention and treatment of *Chlamydia trachomatis*, microbicides	[15]

YWQPYALPL	3 and 8	IL-1R type I	Antagonists	Anti-inflammatory effects	[11]

KRTGQYKL	3	FGFR	Antagonists	Cancer therapy; inhibition of angiogenesis and tumor progression	[18]

GERW**C**FDGPRAWV**C**GWEI, GGNE**C**DIARMWEWE**C**FERL, RGWVEI**C**AADDYGR**C**LTEAQ	8 + 8 and 3 + 3	VEGFR	Indirect antagonists	Cancer therapy; inhibition of angiogenesis and cellular proliferation	[19]

GG**C**ADGPTLREWISF**C**GG	8 + 8	TpoR	Agonist	Treatment of idiopathic	[20]

AFDWTFVPSLIL	3	CCR5	Antagonist	Anti-inflammatory effects, prevention of HIV-1 entry to CD4+ cells; treatment of multiple sclerosis, rheumatoid arthritis, HCV and HIV infections, prevention of renal allograft rejection	[21]

QEVCMTS**C**DKLMK**C**NWMAAM	8 + 8 and 3 + 3	DR5	Agonist	Cancer therapy; triggering of apoptosis in tumor cells	[22]

TAWSEVLDLLRR	3	PMCA4	Allosteric inhibitor	Study of physiological PMCA4 function, study of arterial hypertension mechanisms and retinopathies, development of new class of contraceptives	[23–25]

SS**C**ESPEVDYLE**C**LY, LQ**C**RYDQLIEEWR**C**EY	8 + 8	B-cell maturation antigen	Indirect antagonists	Cancer therapy; inhibition of APRIL (a proliferation-inducing ligand)-stimulated proliferation	[26]
